# The EPIC-26 domain scores after radical prostatectomy are associated with the personality trait of neuroticism

**DOI:** 10.1007/s11255-020-02688-4

**Published:** 2020-10-28

**Authors:** Alv A. Dahl, Sophie D. Fosså, Bjørn Brennhovd, Karol Axcrona

**Affiliations:** 1 National Advisory Unit for Late Effects After Cancer Therapy, Oslo University Hospital, The Norwegian Radium Hospital, Nydalen, P.O. Box 4953, 0424 Oslo, Norway; 2 Faculty of Medicine, University of Oslo, Oslo, Norway; 3 Department of Urology, Oslo University Hospital, The Norwegian Radium Hospital, Oslo, Norway; 4 Department of Urology, Akershus University Hospital, Lørenskog, Norway

**Keywords:** EPIC-26, Neuroticism, Prostate cancer, Robot-assisted laparoscopic prostatectomy, Adverse effects

## Abstract

**Purpose:**

The personality trait of neuroticism represents vulnerability for mental distress to somatic health problems. There are few studies of neuroticism in prostate cancer patients. This study examines the levels of self-reported adverse effects (AEs) after robot-assisted radical prostatectomy (RALP) in Norwegian men with high or low levels of neuroticism. Neuroticism is also compared to relevant factors concerning their associations with various AEs.

**Methods:**

Among 982 men who had RALP at Oslo University Hospital, Radiumhospitalet between 2005 and 2010, 79% responded to a mailed questionnaire in 2011. They rated AEs by completing the EPIC-26 questionnaire, and neuroticism on the Eysenck Personality Questionnaire (EPQ). Men with < 1 year’s follow-up, treatment failure, and incomplete EPQ responses were omitted, leaving 524 men for analysis. The EPQ responses were dichotomized into low and high level of neuroticism. Stepwise multivariate linear regression analyses were used for examination of associations with the EPIC-26 domain scores.

**Results:**

High neuroticism was reported by 20% (95% CI 17–23%) of the patients. On the EPIC-26 dimensions men with high neuroticism had significantly lower mean scores than men with low neuroticism. Most of these between-group differences were clinically significant. In multivariate regression analyses, high neuroticism contributed significantly to all EPIC-26 domains.

**Conclusion:**

Increased levels of AEs after RALP are significantly associated with high neuroticism. A short screening test should be added to the current EPIC-26 instrument to identify patients with high neuroticism. In these patients, pre-operative counseling should take into account their risk of increased AE experiences.

**Electronic supplementary material:**

The online version of this article (10.1007/s11255-020-02688-4) contains supplementary material, which is available to authorized users.

## Introduction

Curative treatments of prostate cancer (PCa) have relatively high rates of well-known adverse effects (AEs) with urinary incontinence and erectile dysfunction being most common after robot-assisted laparoscopic prostatectomy (RALP) [1, 2]. AEs can be registered by health personnel, but in recent years, patient-reported outcome measures (PROMS) have become popular. Among PROMS relevant for men treated for PCa, the Expanded Prostate Cancer Index Composite Short Form with 26 items (EPIC-26) has been recommended [3, 4]. Although psychological factors are of obvious relevance for men treated with radical intention for PCa [5], few studies have examined associations between self-reported AEs and central psychological concepts such as personality. Personality is defined as enduring patterns of perceiving, relating to, and thinking about the environment and oneself. Basic personality traits show considerable stability throughout the life cycle [6]. Modern personality theory mostly describes five basic personality traits (“the big five”) [7], and neuroticism is the most important one concerning health and disease [8].

Neuroticism is the propensity to experience negative emotions, including anxiety, fear, depression, guilt, disgust, irritability, worry, and reduced self-confidence when exposed to stress [9]. High neuroticism predisposes to mental disorders such as depressions [10], anxiety disorders [11], and suicidality [12], reported with increased prevalence in men with PCa. Neuroticism has also been linked to benign pelvic disorders including dysfunction in the urinary and bowel system, explained by the so-called bladder–gut–brain axis [13].

Neuroticism has, however, hardly been studied in men with PCa [14], and particularly not in relation to AEs. In the PCa literature, we found only one prospective study concerning neuroticism and AEs, which documented a significant positive association between neuroticism and sexual bother 1 year after RP [15].

On this background, we wanted to expand the knowledge of the associations between neuroticism, AEs, and other PCa-relevant variables in a cross-sectional sample of Norwegian men treated with RALP. We selected men with ≥ 1 year’s follow-up time after RALP and without treatment failure. Separating that sample into two groups with either high or low neuroticism, we posed two research questions: (1) Are there significant between-group differences of post-treatment on EPIC-26 subs-scale and domain scores? (2) Is high neuroticism significantly associated with the EPIC-26 domain scores post-treatment in forced entry stepwise multivariate linear regression analyses?

## Methods

### Design and ethics

This is a cross-sectional questionnaire-based study of men who previously had RALP at Oslo University. Hospital, The Norwegian Radium Hospital. No corresponding questionnaire was administered before surgery. The study was approved by the Regional Committee for Medical and Health Science Research of South-East. Norway (REK # 2010/1511). All participants have given written informed consent.

### Patients

From January 2005 to August 2010, 988 men underwent RALP at our hospital. By March 2011, a questionnaire was mailed to the 982 survivors, and 777 responded (79% response rate). We omitted 91 men who had follow-up time < 1 year, 146 who reported treatment failure (self-reported as either biochemical recurrence, post-operative radiotherapy, or hormone treatment after RALP), and 16 with incomplete neuroticism data, leaving 524 men as our main study sample.

### Scales

Neuroticism was self-rated on an abridged version of The Eysenck Personality Questionnaire (EPQ) with six items concerning long-term personality characteristics. The items concerned frequent worry, easily hurt feelings, loss of interests, reaction to embarrassing experiences, loss of motivation, and worry that terrible things might happen. Each item is rated as present (1) or absent (0). The sum score ranged from zero to six, and was dichotomized into the high (sum score 3–6) and low neuroticism (sum score 0–2) group as described by the third Health Study of North-Trøndelag County (The HUNT-3 study) [16]. Internal consistency of neuroticism was Cronbach’s coefficient alpha 0.77.

*The EPIC-26* considered AEs of the last 4 weeks covering the urinary, bowel, sexual, and hormonal domains after PCa treatment. The urinary domain included the incontinence and the irritation/obstruction sub-scales. The scores were converted from 0 (worst) to 100 (best) and domain means were calculated [3, 4]. Lower scores reflected increased level of AEs. Cronbach’s alphas were 0.89 for the incontinence and 0.59 for the irritation subscales, 0.77 for the bowel, 0.89 for the sexual, and 0.75 for the hormone domains.

We also defined two category variables based on EPIC-26 scorings: urinary incontinence was defined as no daily use of pads, and erectile dysfunction as inability to perform intercourse.

### Other variables

Risk groups were defined according to D’Amico et al. [17] Clinical and surgical data were collected in a prospective database that has been described previously [18]. Non-paired relation described men who were single or not married/cohabiting. Non-working status concerns men who were without paid work or pensioned. Level of education was dichotomized as short (≤ 12 school) years versus long (> 12 years). Comorbidity was based on self-report, and stroke, diabetes, chronic obstructive lung diseases, liver disease, arthrosis, rheumatic diseases, and kidney disease. We defined three groups: no comorbidity, one comorbid disease, or ≥ 2 comorbid diseases.

### Statistics

Descriptive statistics were performed with chi-square tests for categorical variables and independent sample t tests for continuous variables, and with Mann–Whitney *U* tests in case of skewed distributions. Internal consistencies of measures were tested with Cronbach’s coefficient alpha. According to Osoba et al., we considered between-group differences of > 10% of the total score mean as clinically significant [19].

For the stepwise linear multivariable regression analyses of the EPIC-26 domain scores as dependent variables, four consecutive steps of relevant independent variables were defined: (1) Socio-demography; (2) PCa-related factors; (3) Comorbidity; and (4) High neuroticism. Each step consisted of one to four independent variables with the EPIC-26 sub-scales and domain scores as dependent variables. The contribution of each step to the entire model was calculated as explained variance (*R*^2^), defined as the correlation coefficients squared in percent. The cumulative (total) explained variance of the models was then calculated as the sum of all steps. The strength of the associations between the independent and dependent variables was expressed by B and standardized beta coefficients [20]. The level of significance was set at *p* < 0.05, and all tests were two-sided. Data analyses have been performed with SPSS version 25.0 for PC (IBM, Armonk, NY).

## Results

### Patient characteristics

The mean age at surgery was 62.7 years (SD 5.7), and 22% belonged to the low, 42% to the intermediate, and 36% to the high-risk groups according to D’Amico et al. Twenty-eight per cent of the patients had positive margins, and 72% had bi- or uni-lateral nerve sparing. The mean follow-up time from surgery to survey was 3.0 years (SD 1.5).

### Between-group comparisons on neuroticism

High neuroticism was reported by 107 patients [20% (95% CI 17–23%)] and low neuroticism by 417 [80% (95% CI 76–83%)]. No significant between-group differences were observed concerning the medical PCa-related variables. Significantly more men belonging to the high neuroticism group had short education and more reported comorbidity than the low neuroticism group (Table 1).

**Table 1:** Demographic, clinical, and adverse effect data of the high and low neuroticism groups at survey

Variables	High neuroticism (*N* = 107)	Low neuroticism (*N* = 417)	*p* value	Total sample (*N* = 524)
Age at survey, mean (SD)	64.8 (5.9)	66.1 (5.9)	0.06	65.8 (5.9)
Years RALP-survey, mean (SD)	3.2 (1.2)	3.3 (1.3)	0.19	3.3 (1.3)
D’Amico risk groups, *N* (%)			0.07	
Low	37 (35)	99 (24)		136 (26)
Intermediate	42 (39)	201 (48)		243 (46)
High	28 (26)	117 (28)		145 (28)
Positive margins, *N* (%)	16 (15)	70 (17)	0.65	86 (16)
Nerve sparing, *N* (%)			0.34	
None	30 (28)	91 (22)		121 (23)
Unilateral	49 (46)	196 (47)		245 (47)
Bilateral	28 (26)	130 (31)		158 (30)
Paired relationship, *N* (%)	99 (93)	381 (91)	0.70	480 (92)
Short education, *N* (%)	58 (54)	150 (36)	** < 0.001**	209 (39)
Currently working, *N* (%)	54 (51)	195 (47)	0.47	249 (49)
Comorbidity, *N* (%)			**0.001**	
None	33 (31)	190 (46)		223 (43)
1 disease	39 (36)	154 (37)		193 (37)
≥ 2 diseases	35 (33)	73 (17)		108 (20)
EPIC-26 scores, mean (SD)				
Urinary domain*	69.9 (24.7)	80.2 (20.6)	** < 0.001**	78.1 (21.8)
Incontinence subscale	67.8 (27.5)	74.7 (25.6)	**0.014**	73.3 (26.1)
Irritation/obstruction subscale*	74.0 (29.7)	85.4 (23.4)	** < 0.001**	83.1 (25.0)
Bowel domain*	83.3 (22.2)	93.5 (14.0)	** < 0.001**	91.5 (16.5)
Sexual domain*	27.5 (26.2)	36.3 (28.9)	**0.003**	34.5 (28.6)
Hormonal domain*	66.0 (23.7)	91.7 (15.5	** < 0.001**	86.5 (20.3)
EPIC-26 categories, *N* (%)				
Daily use of pads	42 (40)	142 (35)	0.32	184 (36)
Inability to perform intercourse	89 (83)	302 (72)	**0.023**	391 (75)

^*^Between-groups mean score differences > 10% of total mean score [19]

All EPIC-26 domains and sub-scales showed significantly lower mean scores (higher level of AEs) in the high neuroticism compared to the low neuroticism groups. All between-group domain differences of the EPIC-26 and the urinary irritation subscale were clinically significant [19]. The daily use of pads did not differ significantly between the groups, while inability to have intercourse was significantly more common in the high neuroticism group.

### Stepwise linear multivariable analyses

The multivariable analyses of our four-step regression model with the EPIC-26 domains as dependent variables are displayed in Table 2. The model fits expressed as explained variances (R^2^) varied from 6.6% for the urinary to 29.5% for the hormonal domain. The high neuroticism step made significant contributions to the models for all four EPIC-26 domains. The socio-demographic step made significant contribution to the sexual and hormonal domains, while the PCa-related step only contributed to the sexual domain, and the comorbidity step to the hormone domain.High neuroticism was negatively associated with each of the five EPIC-26 domain scores, and the highest explained variances emerged for the sexual and hormonal domain scores.

**Table 2:** Forced entry multiple linear regression analyses with independent domains (steps) and variables and EPIC-26 domains as dependent variables

Variables	Urinary domain					Bowel domain					Sexual domain					Hormonal domain				
Step	*B*	Beta	*p*	*R*^2^	*p*	*B*	Beta	*p*	*R*^2^	*p*	*B*	Beta	*p*	*R*^2^	*p*	*B*	Beta	*p*	*R*^2^	*p*
1. Socio-demography				0.015	0.10				0.011	0.23				0.131	< **0.001**				0.044	< **0.001**
Increasing age at survey	− 0.32	− 0.09	0.10			− 0.03	− 0.01	0.84			− 1.43	− 0.30	< **0.001**			0.21	0.06	0.18		
Non-paired relationship	− 0.43	− 0.01	0.90			− 1.36	− 0.02	0.60			− 3.05	− 0.03	0.47			− 2.70	− 0.04	0.33		
Short education	− 1.64	− 0.04	0.42			− 0.85	− 0.03	0.58			− 3.81	− 0.07	0.13			− 4.12	− 0.10	**0.012**		
Currently working	− 0.67	− 0.02	0.77			− 2.28	− 0.07	0.20			− 4.24	− 0.07	0.14			− 2.38	− 0.06	0.21		
2. PCa-related issues				0.028	0.33				0.014	0.95				0.167	**0.002**				0.052	0.67
D’Amico risk groups																				
Low (reference)	–					–					–					–				
Intermediate	− 2.69	− 0.06	0.27			− 2.05	− 0.06	0.26			4.56	0.08	0.12			− 1.58	− 0.04	0.42		
High	− 5.11	− 0.11	0.07			− 3.31	− 0.09	0.12			1.09	0.02	0.75			− 4.42	− 0.10	0.053		
Positive margins	2,.53	0.04	0.33			− 0.93	− 0.02	0.64			1.94	0.03	0.54			− 0.49	− 0.01	0.82		
Nerve sparing																				
None (reference)	–					–					–					–				
Unilateral	− 0.01	− 0.00	1.00			− 1.69	− 0.05	0.38			4.85	0.09	0.12			0.15	0.004	0.94		
Bilateral	1.54	0.03	0.62			− 3.30	− 0.09	0.15			8.22	0.13	**0.029**			− 1.64	− 0.04	0.51		
Follow-up time	0.43	0.03	0.58			0.22	0.02	0.71			2.10	0.09	0.029			− 0.67	− 0.04	0.29		
3. Comorbidity				0.033	0.24				0.025	0.054				0.172	0.17				0.080	**0.001**
None (reference)	–					–					–					–				
1 disease	− 2.49	− 0.06	0.25			− 0.19	− 0.01	0.91			0.33	0.01	0.90			− 0.57	− 0.01	0.75		
≥ 2 diseases	− 2.13	− 0.04	0.42			− 3.18	− 0.08	0.11			− 4.32	− 0.06	0.18			− 5.21	− 0.10	**0.015**		
4. High neuroticism				0.066	< **0.001**				0.083	< **0.001**				0.183	**0.009**				0.295	< **0.001**
	− 10.10	-0.19	< **0.001**			− 10.26	− 0.25	< **0.001**			− **7.77**	− 0.11	**0.009**			− 24.37	− 0.48	< **0.001**		
Model explained variance (R^2^)	**6.6%**					**8.3%**					**18.3%**					**29.5%**				

Considering significant single variables, increasing age was negatively associated with the sexual domain score, as was short education with the hormonal domain score. Performance of bilateral nerve sparing RALP and increasing follow-up time were associated with increased sexual domain score, while comorbidity was significantly associated with decreased hormonal domain score.

In Table 3, we have specified the percent contributions by each step since that has to be extrapolated in Table 2. The steps varied from 0.3 to 21.0% of explained variance. High neuroticism showed the highest explained variances for all the EPIC-26 domains.

**Table 3:** Overview of stepwise changes of explained variance (R^2^-change %) for domains and significant variables in relation to EPIC-26 domains and subscales

Domains	Urinary domain		Bowel domain		Sexual domain		Hormonal domain	
Steps	*R*^2^-%	*p*	*R*^2^-%	*p*	*R*^2^-%	*p*	*R*^2^-%	*p*
1. Socio-demography	1.5	0.10	1.1	0.23	13.1	< **0.001**	4.4	< **0.001**
2. PCa-related	1.3	0.33	0.3	0.95	3.6	**0.002**	0.8	0.67
3. Comorbidity	0.5	0.24	1.1	0.47	0.5	0.17	2.8	**0.001**
4. High neuroticism	3.3	< **0.001**	5.8	**0.004**	1.1	**0.009**	21.5	< **0.001**
Explained variance (*R*^2^)	6.6%		9.8%		18.3%		29.5%	

### Sensitivity analysis

The comparisons between the high and low neuroticism groups were also performed in the group reporting recurrences (*N* = 146) (see supplement). The proportion with high neuroticism was 25% (95% CI 18–32%), which did not differ from our main sample (*p* = 0.30). The recurrence sample showed less significant between group differences between the high and low neuroticism groups than the main sample.

## Discussion

Representing a new observation, the results support our hypothesis of a positive association between high neuroticism and decreased levels of EPIC-26 domains scores (at a mean of three years after RALP). In the stepwise multivariate linear regression model, high neuroticism made significant contributions to the total explained variance of all the EPIC-26 domain scores. Taken together these main findings build a strong case for the relevance of neuroticism for self-reported AEs on the EPIC-26 experienced by men treated with RALP.

The sensitivity analyses performed on them patient with recurrence were less convincing concerning these between-group differences. However, the high neuroticism group consisted only of 36 patients representing an increased risk of type II statical errors leading to fewer significant differences.

A major question raised by our results is why high neuroticism has such a strong association with self-rated AEs after RALP. One answer concerns neuroticism as the propensity to experience all sorts of negative emotions and feelings of vulnerability for stress related to the diagnosis of PCa, the treatment, and the development of AEs [8, 9]. Another probable answer is related to the development of the EPIC-26 instrument. The original EPIC-50 instrument covered both function and bother within the different domains. Our research group has previously documented, that the reduction of the EPIC-50 to the EPIC-26 represented preservation of bother items at the cost of functional ones [4]. The Merriam-Webster Dictionary defines bother as “a state of petty discomfort, annoyance, or worry”. [21]. The overlap between bother and neuroticism seems, therefore, obvious since neuroticism is the propensity to experience negative emotions, including anxiety, fear, worry, and reduced self-confidence. The wording of the EPIC-26 items assessing bother rather than function, could contribute to the association between post-RALP AEs and neuroticism.

We included two categorical variables based on the EPIC-26 ratings. Use of pads showed no significant difference between the high and low neuroticism groups. This could be since urine mechanics hardly are associated with high neuroticism. In contrast, the high neuroticism group had a significantly higher prevalence of inability to perform intercourse, which we consider a personality-sensitive issue. The association of increased EPIC-26 bowel domain scores and high neuroticism in our patients puzzles us. Bowel problems as post-RALP AEs have traditionally not been associated with RALP but rather with radiation therapy. We hypothesize that this association is due to the bladder–gut–brain axis having personality as an important factor previously described [13]. Our findings need confirmation in future studies.

Neuroticism as a relatively permanent trait over time is closely related to anxiety and depression as emotional states, eventually as an important etiologic factor for these states [9]. A meta-analysis based on 27 studies demonstrated that the prevalence of anxiety and depression is high in men with PCa [22]. These studies did not include neuroticism as an explanatory or mediating variable, but two studies confirmed the association between high neuroticism and increased levels of anxiety, depression, and mental distress in men with PCa [23, 24]. These observations explain the high contribution of neuroticism to the hormonal domain scores.

Previous studies have focused on the association between post-operative AEs and technical issues of RALP and medical pre-treatment measures, e.g. nerve-sparing and risk groups [25, 26]. These factors are commonly taken into account during the pre-treatment counseling. A consequence of our findings could be that the pre-treatment EPIC-26 should be supplemented not only by the health-related quality of life assessment with the SF-12 scale, but also by a short self-rated screening instrument for neuroticism.

Stepwise linear regression statistics, as performed in our study, compare the contributions of predefined steps of independent variables to outcomes which are the four domains of EPIC-26. The steps selected had their theoretical rationale based on previous research on RALP and AEs. Therefore, we chose relevant socio-demographic variables as our first step, whereas the second step contained relevant medical PCa-related variables. Somatic comorbidity, and high neuroticism also influence AEs after RALP, so they were selected as the next steps. In our statistical model, using the forced entry method, increasing explained variance (*R*^2^) in percent represents better fit of the model [20]. Our model showed good fit for the EPIC-26 hormonal domain (29.5%), and moderate fit for the sexual domain (18.3%), and poor fit for the urinary and bowel domain (6.6–8.3%, respectively). Our explanation is that the urinary and bowel domain scores are more strongly related to local physiological problems than the scores of the sexual and hormonal domains which are highly influenced by increasing age, co-morbidity and high neuroticism.

The prevalence of high neuroticism of 20% in men treated for PCa at our third-line cancer hospital was like the male Norwegian norms of 20% [16]. Our explanation is that a diagnosis of PCa is not associated with increased prevalence of high neuroticism. In this regard, we could expect a significantly increased prevalence of high neuroticism in men reporting recurrence, but so was not the case (see supplement). More significant EPIC-26 differences in our main sample (Table 1) than among men with recurrence (Supplement) could be due to type II statistical error since only 36 men in the recurrence group had high neuroticism.

Finally, we consider the EPIC-26 to be a worthwhile instrument in daily clinical work and for clinical research. Nevertheless, our study reveals the often underrecognized contribution of personality for interpretation of self-reported AEs, particularly for bother issues.

Our data were collected nearly 10 years ago, but we hardly consider that as a relevant issue concerning the scorings of neuroticism. However, our sample concerned the first cohort of men treated with RALP at our hospital. Due to learning curve effects and technical improvements, surgical parameters as proportion of positive margins and of nerve-sparing could have improved in a more recent sample.

Strengths of our study are the considerable sample size, the homogeneity of the sample, and the use of established instruments with good psychometric properties. One limitation of the study is the cross-sectional design. However, since our primary aim was to examine if high neuroticism was significantly associated with the level of AEs after RALP, we consider our cross-sectional design as sufficient. Prospective studies of neuroticism including several treatment modalities are needed to understand the full influence of high neuroticism on AEs after PCa treatment. That the collection of the data was done some years ago, hardly influenced the ratings of AEs and neuroticism.

In conclusion, high neuroticism represents a new, separate, and independent factor influencing the level of self-reported AEs after RALP. High neuroticism, prevalent in about 20% of patients with PCa, contributes to decreasing levels of the EPIC-26 domain scorings. The association between high neuroticism and high levels of AEs, should, therefore, be considered at pre-treatment counseling and when interpreting the patients’ self-report of post-operative AEs.

## Electronic supplementary material

Below is the link to the electronic supplementary material.

Supplementary file1 (DOCX 22 kb)

## Data Availability

According to Norwegian data legislation, the data of this study cannot be made generally available. Requests should be sent to the first author.
