# Supplementary material for: The EPIC-26 domain scores after radical prostatectomy are associated with the personality trait of neuroticism
Source: Int Urol Nephrol. 2020 Oct 28;53(4):691–8. doi: 10.1007/s11255-020-02688-4 (PMC8032572; doi:10.1007/s11255-020-02688-4)
Supplement: Supplementary file 1 — Supplementary file1 (DOCX 22 kb) [file 11255_2020_2688_MOESM1_ESM.docx]

**Supplement**

# Demographic, clinical, and adverse effect data of the high and low neuroticism groups of patients with

| **Variables** | **High neuroticism**  (N = 36) | **Low neuroticism**  (N = 110) | **p-value** | **Total sample**  (N = 146) |
| --- | --- | --- | --- | --- |
| Age at survey, mean (SD) | 64.0 (6.9) | 66.3 (5.2) | 0.07 | 65.8 (5.7) |
| Years RALP-survey, mean (SD) | 3.0 (1.2) | 3.1 (1.3) | 0.76 | 3.1 (1.3) |
| D’Amico risk groups, N (%)  Low  Intermediate  High | 4 (11)  11 (31)  21 (58) | 10 (9)  27 (25)  73 (66) | 0.68 | 14 (10)  38 (26)  94 (64) |
| Positive margins, N (%) | 25 (69) | 82 (75) | 0.55 | 107 (73) |
| Nerve sparing, N (%)  None  Unilateral  Bilateral | 16 (44)  14 (39)  6 (17) | 59 (54)  37 (34)  14 (12) | 0.62 | 75 (51)  51 (35)  20 (14) |
| Paired relationship, N (%) | 34 (95) | 104 (94) | 0.98 | 138 (94) |
| Short education, N (%) | 17 (47) | 41 (37) | *0.29#* | 58 (40) |
| Currently working, N (%) | 17 (47) | 43 (39) | 0.39 | 60 (41) |
| *Comorbidity, N (%)*  None  1 disease  ≥ 2 diseases | 15 (42)  12 (33)  9 (25) | 46 (42)  40 (36)  24 (22) | *0.91* | 61 (42)  193 (37)  108 (20) |
| *EPIC-26 scores, mean (SD)* Urinary domain*  Incontinence subscale  Irritation/obstruction subscale*  Bowel domain  Sexual domain*  Hormonal domain* | 58.1 (27.4)  52.2 (34.3)  70.7 (24.8)  81.5 (23.1)  20.2 (26.8)  57.5 (22.0) | 71.0 (22.3)  62.7 (28.8)  81.1 (22.7)  86.9 (20.3)  25.9 (23.1)  81.0 (23.9) | **0.005**  *0.07*  **0.021**  *0.18*  *0.22*  **<0.001** | 67.8 (24.2)  60.1 (30.4)  78.5 (23.6)  85.5 (21.1)  24.5 (24.1)  75.2 (25.5) |
| *EPIC-26 categories, N (%)*  Daily use of pads  Inability to perform intercourse | 20 (55) 28 (78) | 58 (53) 93 (85) | 0.77 *0.35* | 78 (53) 121 (83) |

**reported relapse (N = 146).**

*Between-groups mean score differences > 10% of total mean score [19]. # Italics means significant between-group differences in our main sample (N = 524)
